# Editorial: Forensic investigative genetic genealogy and fine-scale structure of human populations

**DOI:** 10.3389/fgene.2022.1067865

**Published:** 2023-01-05

**Authors:** He Guanglin, Wei Lan-Hai, Wang Mengge

**Affiliations:** ^1^ Institute of Rare Diseases, West China Hospital of Sichuan University, Sichuan University, Chengdu, China; ^2^ School of Ethnology and Anthropology, Inner Mongolia Normal University, Hohhot, China; ^3^ Faculty of Forensic Medicine, Zhongshan School of Medicine, Sun Yat-Sen University, Guangzhou, China

**Keywords:** population structure, admixture history, genome-wide genetic markers, forensic investigative genetic genealogy, genetic diversity

## Introduction

Anatomically modern humans originated in Africa and separated from their most likely recent common ancestor hundreds and thousands of years ago (Bergstrom et al., 2020; Wang et al., 2021a). They followingly migrated out of Africa around 50 thousand years ago and evolved in concert with the complicated interplay of gene flow and adaptive selection during the peopling of Eurasia, Oceania, and America (Bergstrom, et al., 2020; Wang et al., 2021a). Genomic studies have demonstrated the pervasiveness of population differentiation and genetic admixture between long-isolated ethnic groups (Bergstrom et al., 2020; Pan et al., 2022). Extensive population bottleneck, adaptive evolution in changing environments, and introgression from archaic hominins further shaped the complicated patterns of human genetic heritage. In general, complex population divergence, migration, and admixture events extensively shaped the patterns of genetic diversity of ethnolinguistically diverse populations.

There is increasing evidence to suggest that the differences in the susceptibility of many common and rare diseases are primarily attributed to human populations’ diverse cultural, environmental, demographic, and genetic histories. The comprehensive understanding of fine-scale population evolutionary history will gradually change our understanding of the genetic architecture of diseases (Timpson et al., 2018; Benton et al., 2021; Pan et al., 2022). Thus, there is an urgent need to expand genetic research to populations with different ancestries. In addition, population genetic studies based on multiple genome-wide genetic markers (short tandem repeat, STR; single nucleotide polymorphism, SNP; Insertion/Deletion, InDel; copy number variation, CNV, and so on) could provide new insights into the detailed process of population admixture and evolutionary history of ethnolinguistically and geographically diverse populations. Understanding the fine-scale population structure also helps the better study design in medical genomics and the comprehensive practice applications in population genetics and forensic science.

With the rapid development of genotyping technologies, sequencing platforms, and computational methods, previous studies have provided the basal framework of the genetic landscape of worldwide populations from different perspectives (Li et al., 2008; Lippold et al., 2014). However, most genetic studies focused on the relationships and fine-scale population structures were conducted *via* low-density genetic markers. It is also now possible to capture and sequence ancient DNA from ancient samples, which could provide pivotal insights into the formation of spatiotemporally diverse populations with unprecedented resolution. Although huge nationwide biobanks for characterizing the genotypes and phenotypes of millions of people have been established (Barton et al., 2021; Zhang et al., 2021; Chiu et al., 2022), more geographically, linguistically, and culturally diverse populations (especially non-metropolitan populations) are needed to be studied systematically at different spatio-temporal scales.

A clear understanding of genetic background and diversity of ethnolinguistically diverse populations and decoding their demographic history can provide new medical and forensic application opportunities. Genetic studies have illuminated the population-specific reference database, effective algorithm and the developed panel for the targeted forensic applications were the fundamentals of forensic intelligence inference of external visual appearance, biogeographical ancestry inference and forensic investigative genetic genealogy (FIGG) (He et al., 2018). FIGG, one new and rapidly growing field of forensic genetics since 2018, has attracted the attention of geneticists focused on complex familial search (Phillips 2018). Currently, many projects aim to develop and validate new FIGG panels, construct and complement forensic FIGG databases, and develop new statistical models to promote the practice of FIGG (Kling et al., 2021; Tillmar et al., 2021).

To summarize the new advances in FIGG and fine-scale population structure and illuminate the importance of full-scale genetic structure and diversity as the basis for the FIGG, we organized this Research Topic of the “*Forensic investigative genetic genealogy and fine-scale structure of Human Populations*”. In detail, this Research Topic aimed to characterize the genetic background and demographic history of ethnolinguistically and geographically diverse populations based on different densities of genetic marker. It would be helpful in exploring the long-range familial searches and fine-scale genetic localization within subgroups in other continents. This Research Topic attracted research focused on the basic knowledge exploration of the genetic background of one targeted population and included applied research focused on developing and validating forensic amplification systems.

## Exploration of theoretical knowledge—Fine-scale genetic structure and demographic history reconstruction

Recent studies have demonstrated that population history reconstruction leveraging high-density genetic markers could uncover previously unrecognized population structures at a fine scale compared with forensically relevant loci (Li, et al., 2008; Bergstrom, et al., 2020). Zhou et al. generated and analyzed genome-wide data of Liaoning Han people and found that genetic differences existed in geographically different Sinitic-speaking Han populations, which might result from other migration and admixture events of Hans during the period of “Chuang Guandong”. Hou et al. investigated the population history of Liaoning Mongolians based on ∼700,000 SNPs and provided new insights into the admixture history of Mongolic-speaking Mongolians according to shared allele-based analyses. He et al. explored the demographic history of Qiang people based on Eurasian modern and ancient reference populations. This study revealed that the Tibeto-Burman-speaking Qiang people derived their primary ancestry from Tibetan-related ancestral populations in North China. Wang et al. performed a genome-wide association study on 26,806 Chinese individuals. They identified 21 SNPs associated with widow’s peak, unibrow, double eyelid, earlobe attachment, and freckles. This study may facilitate a better understanding of the genetic basis of facial development in Chinese populations and provide new markers for forensic phenotype predictions. These studied Han, Mongolian and Qiang people are widely distributed in North China, and other populations from southern China and surrounding regions need to be further explored based on the array or sequencing data, such as Austronesian, Austroasiatic, Hmong-Mien and Tai-Kadai people. Generally, population genetic studies showed a strong correlation between Chinese cultural language and geographic patterns and population structure.

The estimated patterns of genetic diversity in China can help accurately biogeographic ancestry inference and FIGG. Similar population stratifications were also identified in Siberian populations. Currently, available tools could effectively distinguish populations with different continental origins, but most of these are not efficient for differentiating people within the same continent. Gorin et al. selected 5,229 AISNPs and tested various mathematical models for biogeographic ancestry inference. The results showed that the accuracy of the prediction of this panel on one of 29 studied ethnic groups reached 71% and the proposed method could be employed to predict ancestries from Russian and neighboring populations. Huang et al. developed a machine learning approach for estimating the relationships with high error SNP profiles and found that this approach was more accurate and robust than the individual measures.

## Forensic potential applications—Development and validation of forensic systems focused on personal identification and family research

STR genotyping has been applied in forensic investigations for nearly 30 years (Hagelberg et al., 1991; Kayser and de Knijff 2011). Nowadays, several commercial STR kits have been developed based on the expanded CODIS loci (Oostdik et al., 2014; Wang et al., 2018; Qu et al., 2019; Batham et al., 2020; Green et al., 2021). However, with the rapid increase in the number of STR genotypes in forensic databases, more novel non-CODIS STRs with high genetic polymorphisms are required to minimize the incidence of adventitious matches. Huang et al. validated the forensic performance of a novel multiplex autosomal STR panel (including six CODIS STRs and 20 non-CODIS STRs). They found that this novel kit could be applied as a promising tool for forensic human identification and complex paternity analysis. Than et al. genotyped seven Lao Isan and three Laotian populations using Verifiler plus PCR Amplification kit. The allelic frequency results provided the genetic background of Austroasiatic and Tai-Kadai people from Laos and Thailand.

InDel loci, the second most abundant polymorphism across the human genome, possess low mutation rates and small amplicon lengths compared with STRs (Weber et al., 2002; Mills et al., 2006), which have been proven to be of value in forensic investigations (Pereira et al., 2009; Zhang et al., 2018). And InDels showing significant allele frequency differences among geographically and linguistically diverse populations can be adopted as ancestry-informative markers (AIMs) (Sun et al., 2016; Inacio et al., 2017). Moreover, previous studies showed that multi-InDel loci behaved well in parentage tests and could be used for forensic applications (Fan et al., 2016; Sun, et al., 2016; Qu et al., 2020). Liu et al. developed and validated a six-color fluorescence multiplex panel including 59 autosomal InDels. Subsequently, Tibetan groups from China have been genotyped using the newly-developed 59-plex InDel panel. The comprehensive population genetic analyses showed that this homemade panel could be used as a powerful tool for individual forensic identification and paternity testing in Chinese Tibetan groups. Jin et al. developed a Next-Generation Sequencing (NGS) InDel panel, including 17 multi-InDels on the X chromosome. They found that the newly-developed panel could be adopted as an effective tool for individual forensic identification, paternity testing, and biogeographical ancestry inference.

Genetic surveys based on uniparentally inherited markers have identified many paternal and maternal founding lineages in regional populations and their corresponding expansion events (Poznik et al., 2016; Li et al., 2019). Jia et al. sequenced complete mitochondrial genomes of 146 Daur individuals in China. The results showed that the Daur ethnic group has high maternal genetic diversity and may have experienced recent population expansion. He et al. developed and validated the AGCU-Y30 Y-STR panel and conducted a Y-STR-based study to explore the paternal history of the Qiang people. The validated results showed that the novel Y-STR kit was sensitive and robust enough for forensic applications. Population genetic analyses revealed that the Qiang people are closely related to lowland Tibetan-Yi corridor populations.

## Prospects and challenges

Human genetics needed the fundamental foundation supporting large-scale population genomic projects to characterize the full landscape of human genetic diversity and population structure, such as NHLBI TOPMed (Taliun et al., 2021), gnomAD (Collins et al., 2020) and UK10K (Wang et al., 2021). These projects made significant advances in European human genetics. Other projects, including the Chinese 10K_CPGDP (Chinese population genomic diversity project), GSRD-100K^WCH^ and ChinaMap, were recently launched to explore the genetic features of under-represented populations. Characterizing the genetic architecture of ethnolinguistically and geographically diverse populations will promote our understanding of population origin, separation, admixture, adaptation, and gene flow from archaic individuals.

Knowledge of the fine-scale genetic structure and population-specific genomic database is the base for FIGG. Synthesizing our growing knowledge of evolutionary history with forensic investigations will help to achieve the promise of long-range familial searches and fine-scale genetic localization. For human population genetics and FIGG, we also had some challenges that needed to be overcome in the next step:

Firstly, European bias in worldwide human genetic studies and Han Chinese bias in Chinese cohort research hinder in heath equality of genetic studies and forensic genetics. More population genomic studies from under-reported populations need to be conducted to characterize their uncharacterized genetic polymorphisms and the genetic spectrum of different genetic markers.

Second, a population-specific genomic database should be constructed to provide a comprehensive landscape of different genetic markers and even include structural variations and mobile elements via the PacBio sequencing plateau.

Third, cooperation of national genomic studies should be formed to promote data sharing in different institutions.

Forth, population genomic projects should be conducted among included subjects with deep phenotypes, which would provide values for exploring the genetic basis for physical traits and medical phenotypes.

Fifth, regional population-specific genomic datasets, FIGG panels, algorithms, and forensic databases should be developed and validated. FIGG has promoted successful inspection of criminal suspects among American or European populations, as there are publicly available European/American genomic databases and DNA. Land and GEDmatch severs. Similar databases from other countries or regions should be constructed in the future.

Seventh, IBD-based algorithms need the high-density phased SNPs or whole genome sequencing data. Many forensic samples were highly degraded or had minute amounts of genetic materials. Thus, the primary focus is to develop more FIGG panels based on low-density SNP markers and non-linkage algorithms (allele-sharing status and allele frequency spectrum).

In short, more ethnolinguistically and geographically diverse populations are needed to be studied based on different genetic markers and algorithm models, and population-specific panels based on different genetic variations and corresponding forensic databases need to be developed to achieve long-range familial searches and fine-scale genetic structure reconstruction.
